# Evolutionary Dynamics of Satellite DNA Repeats across the Tettigoniidae Family: Insights from Genomic Analysis

**DOI:** 10.3390/biom14080915

**Published:** 2024-07-27

**Authors:** Muhammad Majid, Hashim Khan, Xuanzeng Liu, Muhammad Shaheer, Yuan Huang

**Affiliations:** 1 College of Life Sciences, Shaanxi Normal University, Xi’an 710119, China; majidento07@snnu.edu.cn (M.M.);; 2 Department of Entomology, MNS Agriculture University, Multan 66000, Pakistan

**Keywords:** satellite DNA, transposable elements, Tettigoniidae, chromosomal distribution, evolutionary trajectories

## Abstract

Satellite DNA repeats are repetitive DNA sequences found in eukaryotic genomes, typically consisting of short DNA motifs repeated in tandem arrays. Despite the vast body of literature on satellite DNA repeats in other taxa, investigations specifically targeting Tettigoniidae remain conspicuously absent. Our study aims to fill a critical gap in our understanding of satellitome evolutionary processes shaping Tettigoniidae genomes. Repeatome analysis revealed that the *Meconema thalassinum* genome comprises 92%, and *Phryganogryllacris superangulata* had the lowest value of 34%, with an average of 67% in other Tettigoniidae species. The analysis reveals significant variation in the number of satellite DNA repeats across species of the Tettigoniidae family, with *M. thalassinum* exhibiting the highest count, 246, reported in insects to date and the lowest count, 10, in *Pholidoptera griseoptera*. *Ruspolia dubia* and *Ruspolia yunnana*, which are congeneric species, showcase distinct counts of 104 and 84 families, respectively. Satellite DNA repeats in *R. dubia* exhibit the highest abundance, constituting 17.2% of the total genome, while the lowest abundance was reported in *P. griseoptera*, at 5.65%. The genome size correlates weakly with the satellite DNA family count (rs = 0.42, *p* = 0.29), but a strong correlation exists between satellite abundance and family number (rs = 0.73, *p* = 0.03). Moreover, the analysis of satellite DNA gain and loss patterns provides insights into the amplification and homogenization of satellite DNA families within the genome, with species-specific repeats exhibiting a positive trend toward amplification. The chromosomal distribution in *M. thalassinum* displayed that the highest accumulation was observed on Chr12, Chr01, and Chr04, constituting 17.79%, 17.4%, and 17.22% of the total chromosome size, respectively. The chromosome-specific propagation of satellite DNA families was evident, with MthSat01 solely on chromosome 1 and MthSat170 on chromosome 2, sharing 1.64% and 2.33%. The observed conservation and variations in satellite DNA number and abundances, along with distinct patterns of gain and loss, indicate the influence of potentially diverse evolutionary processes shaping the genomic landscape of these insects, which requires further investigation. Furthermore, the differential accumulation of satellite DNA on specific chromosomes implies that potential chromosome-specific functions or structural features influence the retention and proliferation of satellite sequences.

## 1. Introduction

Satellite DNAs and transposable elements (TEs) in insects exhibit both conservation and variations in their sequences [1,2]. Satellite DNA sequences, characterized by their tandemly repeated units, play crucial roles in genome organization and evolution [3,4,5]. Orthoptera species are noteworthy in satellite DNA research due to the notably higher abundance of satellite DNA repeats within their genomes. This abundance distinguishes them from other insect orders, where satellite DNA repeats are comparatively less prevalent [6,7]. Satellite DNA repeats vary in length among species and are categorized as either simple or complex based on their repetitive unit size [8]. The satellite DNA distribution can be species-specific, as demonstrated in studies on *Gryllus bimaculatus* [9], Gomphocerine grasshoppers [10], different *Drosophila* species [11], and locusts [12,13], though some variants are shared among related species. Likewise, the chromosomal distribution reflects that satellite DNA repeats are prevalent components of centromeres, which is crucial for assembling centromeric chromatin [14,15,16]. Similarly, satellite DNA families can often be shared among related species, with one predominant family present in each species across the total satellite DNA library. This pattern is observed in different species of the family Tenebrionidae, where one or two families are predominant [17,18,19]. Similar observations have been made in *Tribolium castaneum* [20] and in the genus Schistocerca [21].

Tandemly repeated (satellite DNA) sequences exhibit two primary characteristics: low sequence variability within repeat units and significant variability in copy numbers [22,23,24]. The concept of concerted evolution [25] suggests that monomers within arrays of satellite DNA evolve collectively, maintaining low sequence variability. As a result, while the homogeneity of satellite DNA is preserved within species, the DNA sequence among separate groups of individuals is expected to diverge rapidly. This divergence is driven by the homogenization and fixation of different mutations in the monomers of each group, ultimately leading to the formation of species-specific variants [4,10,12,26,27,28]. 

The whole set of satellite DNA sequences, referred to as the satellitome, varies greatly across different species. For instance, in the moth *Cydalima perspectalis*, only one satellite DNA sequence was identified, constituting approximately 0.14% of the genome [6]. In contrast, a higher diversity of satellite DNA sequences was found in grasshopper species, with 129 sequences detected in the morabine grasshopper [29] and 62 in the migratory locust [30]. The ladybird beetle *Hippodamia variegate* harbors 30 satellite DNA sequences, accounting for 15% of its genome [31]. Similarly, the kissing bug *Rhodnius prolixus* possesses 39 satellite DNA sequences, comprising 8% of its genome [32]. In vertebrates, the fish *Megaleporinus microcephalus* harbors 164 satellite DNA sequences, with the most abundant representing 2.78% of its genome [33]. The freshwater crab *Pontastacus leptodactylus* exhibits the highest number of satellite DNA sequences, totaling 258, which collectively constitute approximately 28% of its genome [34]. The satellitome of *Triatoma delpontei* comprises 160 satellite DNA families, collectively constituting significant portions, approximately 18%, of the insect genome [35]. Drosophila, extensively studied in terms of the satellitome, has been examined across 58 species, resulting in the characterization of numerous satellite DNA sequences [11,36]. The diversity in the number of satellite DNA sequences and their contribution to genome size across different species highlights the dynamic nature of genomic organization. It suggests that the abundance and composition of satellite DNA can vary widely among organisms, even within the same taxonomic group. This diversity may reflect species-specific evolutionary histories, ecological adaptations, or genomic structural constraints. 

In the present study, we selected eight species from the orthopteran insect family Tettigoniidae, each exhibiting varying levels of genome size. Additionally, one species from the Gryllacrididae family, which diverged from Tettigoniidae approximately 175 million years ago, was chosen as an outgroup. A comprehensive comparative analysis of satellite and transposable element (TE) repeats was conducted to investigate the correlation between genome size and satellite DNA repeat number across species within this highly diverged group. The genome of the Tettigoniidae family consists of a higher percentage of transposable elements (TEs), ranging from 92% in the *M. thalassinum* species to lowest 34% in *P. superangulata*. Additionally, the satellitome percentage varies from 17.2% in *R. dubia* to 5.65% in *P. griseoptera*. Interestingly, there is no correlation between genome size and the number of satellite DNA families within the genome. The higher percentage of satellitome within Tettigoniidae underscores the importance of conducting in-depth analyses of additional species to examine its role in the genome structure and architecture.

## 2. Materials and Methods 

### 2.1. Sample Collection, Next-Generation Sequencing, and NCBI Data Retrieval

Samples from three species of the Tettigoniidae family, *A. sinensis*, *R. dubia*, and *M. bonneti* were collected from various regions in China, while the genome data of six additional species, including *P. superangulata*, *E. pallidus*, *R. yunnana*, *P. griseoaptera*, *G. gratiosa*, and *M. thalassinum*, were downloaded from the SRA NCBI database. The genome assembly of M. thalassinum species was also obtained for this analysis. The freshly collected samples were preserved at −80 degrees Celsius to ensure their viability for subsequent DNA extraction and genomic analysis. The genome size of the lab species was estimated using flow cytometry following standard protocols. Later, these samples were sent for sequencing using the Illumina sequencing platform with a paired-end read length of 150 bp and an insertion library of 350 bp. Complete details of the species and their respective genomic data SRA numbers are provided in the supplementary table (refer to Table S1 for details).

### 2.2. Pre-Processing of the Genomic Data

In this study, we utilized the WGS (Whole Genome Shotgun) data from each species for analyzing satellite DNA and transposable elements (TEs). We adhered to the recommended optimal genome coverage for the TAREAN tool, which falls within the range of 0.01–0.5×. To ensure comprehensive genome representation, we used the SeqTK v1.4 tool (https://github.com/lh3/seqtk, accessed on 19 October 2023) for random sampling. From each sample, we randomly extracted 3 million reads for tandem repeat analysis. The data were uploaded to the lab server, and their quality was assessed using the command-line version of FastQC v0.12.0 on Linux. We pre-processed the fastq files with the Trimmomatic tool, which involved trimming, quality-filtering the reads, discarding single reads while retaining complete pairs, and performing cut-adapt filtering. After quality filtering, we used the reformat.sh script to interlace the two quality-filtered fastq files.

### 2.3. RepeatExplorer2 and TAREAN Clustering Analysis

We performed repeat clustering analysis using the command-line versions of TAREAN and RepeatExplorer2 (Biology Centre AS CR, Ceske Budejovice, Czech Republic), utilizing the interlaced FASTA file generated in the previous step (http://repeatexplorer.org/?page_id=818, accessed on 2 December 2023). For RepeatExplorer2 clustering, we set the parameters as follows: paired-end reads = yes, sample size = 3 million reads, reference database = Metazoa version 3.0, and custom database = Repbase in advanced options. For TAREAN, we used the default settings with a sample size of 3 million reads and added the “-t” parameter to run in TAREAN mode only. The clustering analysis produced three output files: a log file, an HTML report, and an HTML archive report, which were examined for further analysis. We manually inspected the HTML report, which contains the annotation of repeatome, including satellite DNA repeats. The total percentage of repeatome was calculated by summing the reads reported in the different clusters. The number of satellite DNA families is reported after manual curation, which involved removing mitochondrial sequences and other multi-gene families. The total number of satellite DNA repeats includes both high-confidence and low-confidence families obtained from TAREAN clustering. We confirmed the satellite DNA repeats by manually inspecting the clusters. Clusters with ring or spherical shapes were counted, and further confirmation, where necessary, was performed by subjecting the contigs of relevant clusters to the YASS v1.15 tool for dot plots. All satellite DNA family consensus sequences were concatenated for each species’ satellite DNA family in accordance with the proposed nomenclature by Ruiz et al. [30]. The correlation between repeatome and genome sizes, as well as between the number of satellite DNA repeats and genome size, was assessed using Spearman correlation test.

### 2.4. Satellite DNA Homology Search and RepeatMasker Analysis

To classify satellite DNAs into superfamilies based on homology, we performed all-to-all comparisons using the ‘rm_homology.py’ script from the satminer toolkit (https://github.com/fjruizruano/satminer, accessed on 18 January 2025). Additionally, we used the Censor tool (http://www.girinst.org/, accessed on 19 January 2024) to search for homology between each satellite DNA and the existing transposable elements (TEs) in the Repbase database. We first examined homology using the arthropods section of Repbase and then searched all databases for similarities to satellite DNA consensus sequences. We also investigated any similarity or coding sequences in each satellite DNA family against the Dfam database and NCBI nucleotide databases using BLAST.

We then merged the consensus sequences of satellite DNA repeats from all species to create a Tettigoniidae family library for downstream analysis. Using RepeatMasker v4.1.6 (http://repeatmasker.org, Seattle, USA, accessed on 11 February 2024) with the “-a” option, custom library (-lib), and the NCBI BLAST search engine, we profiled the divergence and abundance of each satellite DNA repeat against each species. The interlaced FASTA file was used as input for RepeatMasker to align against the complete library of satellite DNA consensus sequences with the customized reference library option. The average divergence of each satellite DNA repeat from its consensus sequences was calculated using the “calcDivergenceFromAlign.pl” script. This step generated a divsum file that contains absolute abundance and divergence values for each satellite DNA family. This divsum file was converted into a CSV file to generate a satellitome landscape using Python scripts. To evaluate the overall gain and loss of each satellite DNA, we calculated standardized Z-score values of abundance and divergence in an Excel spreadsheet. The overall gain for satellite DNA repeats within the genome is considered when the z-score abundance values are positive and the z-score divergence values are negative. The overall loss is considered for a specific satellite DNA family when the z-score abundance value is negative, irrespective of the z-score divergence values.

## 3. Results

### 3.1. Repetitive Sequences Composition and Distribution

**Figure 1:**
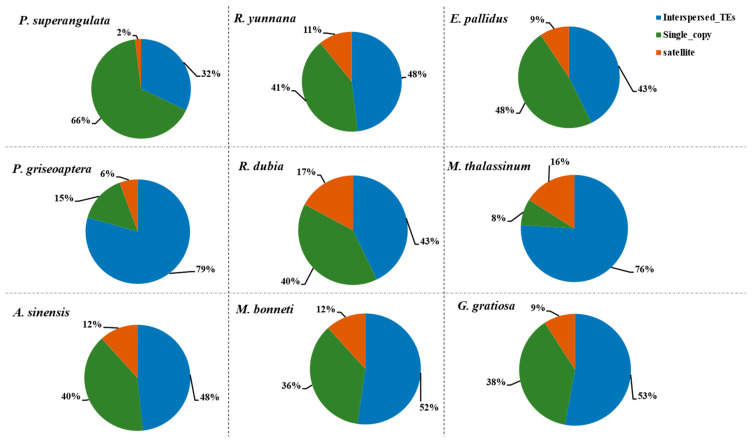
Satellite DNA and interspersed TE distribution across nine species. The pie charts depict the overall composition of total genomes, categorizing them into interspersed TEs, satellite DNA repeats, and single-copy sequences.

We divided the whole genome composition into two categories: repeatome and unique sequences. We discovered that the genome of *M. thalassinum* consists of almost 8% unique sequences and 92% repeatome, with 76% being interspersed repeats and 16% satellite DNA repeats. Contrastingly, the outgroup species *P. superangulata* exhibited the lowest percentage of repeatome, at 34%, with 7% comprising satellite DNA repeats. The genome of *P. griseoaptera* predominantly shared 85% repeatome and 15% single-copy sequences. Further exploration into congeneric species *R. dubia* and *R. yunnana* unveiled genomes composed of 60% and 59% repeatome, respectively. Notably, there were differences in satellite DNA abundance, with *R. dubia* at 17% and *R. yunnana* at 11%. The other species, *E. pallidus*, *A. sinensis*, *M. bonneti*, and *G. gratiosa*, constitute 52%, 60%, 64%, and 62% of the total genome, respectively (see Figure 1). We did not observe any significant correlation between genome size and repeatome in the Tettigoniidae family (Pearson correlation, rs = 0.08). In the Tettigoniidae species, repeatome content ranged from 52% to 92%, with an average of 67%. However, in the outgroup species, repeatome abundance exhibited a notable decrease to 34%. This decrease in TE content in the outgroup species suggests potential differences in TE regulation or evolutionary dynamics compared to the Tettigoniidae species. We found no correlation between genome size and the abundance of repeatome within Tettigoniidae species (coefficient rs = 0.08, *p*-value = 0.84). The satellite DNA abundances discovered in this study varied significantly, ranging from a minimum of 5.65% to a maximum of 17.2%, with an average of approximately 11% across the nine species examined. These findings underscore the variability in satellite DNA content among the species studied.

### 3.2. Satellite DNA Repeat Diversity in Family Tettigoniidae

**Figure 2:**
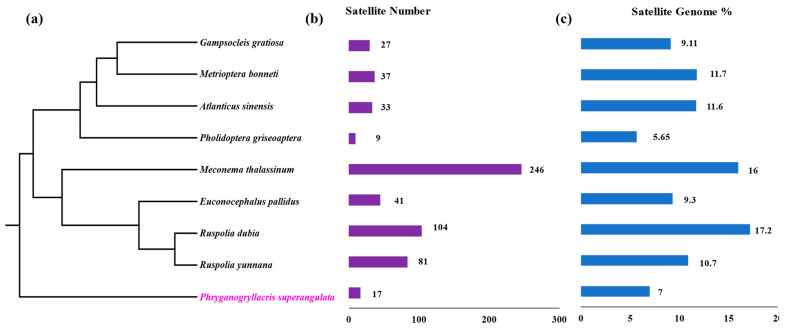
Satellitome diversity across species of the Tettigoniidae family. (**a**) Phylogenetic relationships among eight species of the Tettigoniidae family, with *P. superangulata* as the outgroup. The divergence time from the family Tettigoniidae is ∼175 Ma. This phylogeny was based on the mitochondrial genome. (**b**) The number of satellite DNA repeats across different species. (**c**) The percentage of the genome occupied by satellite DNA repeats among different species.

The satellite DNA repeats exhibited great diversity in terms of the total identified number across the Tettigoniidae family. The highest number of satellite DNA repeats was observed in *M. thalassinum* species, with 246 satellite DNA repeats, marking the highest count reported in insect species to date. The minimum was identified in Pholidoptera griseoptera species, with only 10 satellite DNA families. *E. pallidus*, closely placed on the current phylogenetic tree with *M. thalassinum*, possesses 45 satellite DNA families. Similarly, closely related species on the phylogenetic tree—*G. gratiosa*, *M. bonneti*, and *A. sinensis*—show less variation in total satellite DNA families, with counts of 30, 37, and 33 respectively. The congeneric species *R. dubia* and *R. yunnan* have different numbers of satellite DNA families, with 104 and 84, respectively (Figure 2b). The species *P. superangulata*, used as an outgroup, has 17 satellite DNA families, constituting only 7% of the whole genome, which is less than the average that observed in Tettigoniidae species.

Satellite DNA repeats in *R. dubia* exhibit the highest abundance, constituting 17.2% of the total genome, while the lowest was reported in *P. griseoptera*, at 5.65%. The sister species *R. yunnana* contributes 10.7% of the total genome. Interestingly, *M. thalassinum*, despite having the highest number of satellite DNA repeats, ranks second in terms of constituting 16% of the total genome. The closely linked species *G. gratiosa*, *M. bonneti*, and *A. sinensis* share fractions of 9.11%, 11.7%, and 11.6%, respectively, of the total genome. Overall, species of the Tettigoniidae family exhibit higher satellitome abundance and diversity compared to the outgroup species *P. superangulata* (see Figure 2c). We did not observe any correlation between genome size and the number of satellite DNA families (coefficient rs = 0.42, *p*-value = 0.29), but we observed a strong correlation between satellite abundance and the number of satellites within the genome; the higher the number, the higher the abundance (coefficient rs = 0.73, *p*-value = 0.03).

### 3.3. Satellite DNA Gain and Loss in Family Tettigoniidae

**Figure 3:**
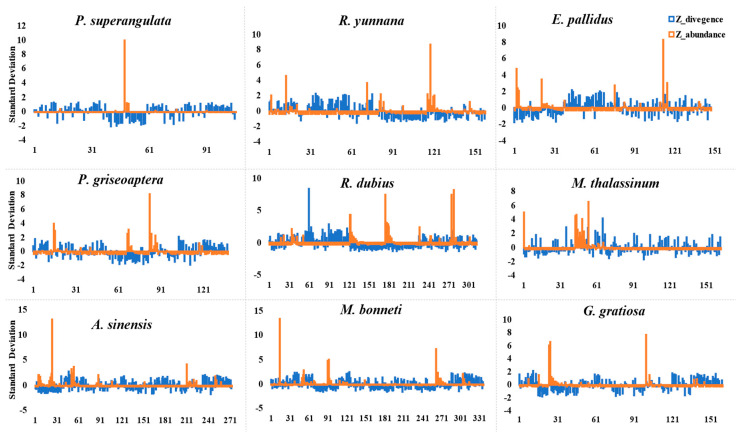
Overall gain and loss of satellite DNA families in nine species. Z-score abundance and divergence were calculated for each satellite DNA family in each species. The x-axis depicts the total satellite DNA repeats (including species-specific, older residual copies from other species, and fragmental copies), while the y-axis shows the number of standard deviations from the mean value.

We calculated the z-score abundance and divergence of satellite DNA repeats to observe the overall homogenization, gain, and loss patterns across the Tettigoniidae family in comparison to outgroup species. Here, we assess gains for satellite DNA families with positive z-score values and negative z-score divergence, as well as gains with positive z-score abundance and positive z-score divergence and losses with negative z-score abundance. We found that species-specific satellite DNA repeats exhibited positive trends toward the amplification within the genome compared to older conserved satellite DNA repeats. Notably, the *R. dubia* genome demonstrated the highest gain of 54 satellite DNA, and the lowest gains were observed in the outgroup species *P. superangulata*, with eight satellite DNA repeats gained. The second-highest gain was observed in *M. bonneti*, with 50 satellite DNA repeats, and 8 were highly abundant. Similarly, *A. sinensis*, *R. yunnana*, *E. pallidus*, *P. griseoptera*, and *G. gratiosa* exhibited gains of 47, 36, 28, 26, and 28 satellite DNA families, respectively (Figure 3 and Figure S1).

Interestingly, *M. thalassinum*, despite being the second-highest in abundance, showed gains for only 23 satellite DNA families, the lowest among tettigoniidae species. We further sorted out highly abundant satellite DNA families with a z-abundance above 1 and a negative z-divergence, indicating homogenization patterns within the genome. In *R. dubia*, we identified 14 highly abundant satellite DNA families, while *P. superangulata* species had 3. *M. thalassinum* and *A. sinensis* each possessed seven satellite families, and *R. yunnana*, *M. bonneti*, and *G. gratiosa* each had six. The genomes of *E. pallidus* and *P. griseoaptera* contained four and five highly abundant satellite DNA repeats, respectively (see Figure S2). This suggests that certain satellite repeats were tolerated within the genome and allowed to propagate, while others were excluded over time.

### 3.4. Satellitome Landscape of Species of the Tettigoniidae Family

**Figure 4:**
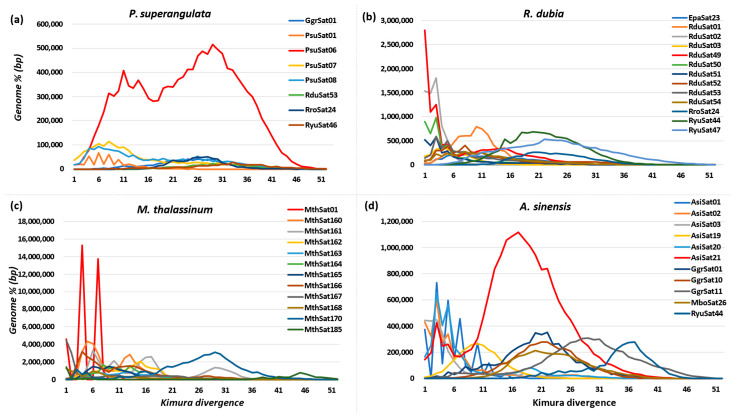
Satellitome landscape of species of the Tettigoniidae family. (**a**) Satellite DNA repeat landscape for *P. superangulata* species. (**b**) Satellite DNA repeat landscape for *R. dubia* species. (**c**) Satellite DNA repeat landscape for *M. thalassinum* species. (**d**) Satellite DNA repeat landscape for *A. sinensis* species.

We constructed a satellitome landscape of highly abundant satellite DNA families across all nine species and described those with an abundance exceeding 0.2%. The divergence rate from the consensus sequence ranged from a minimum of 1.02% to a maximum of 43% in *R. dubia*, with a median value of 12.3%. Similarly, descriptive statistics for all species’ divergence and abundance are presented in the Supplementary Information (see Tables S2–S10). The *P. superangulata* genome possesses three highly abundant satellite repeats, with the PsuSat06 satellite family constituting 4% of the total genome (Figure 4a). The genome of *R. dubia* harbors 13 highly abundant satellite DNA families, with a recent peak in the landscape for species-specific rather than older bursts from other species satellite families. The RduSat49 family constituted 1.25% of the total genome, and although we did not observe a recent burst for the RyuSat44 and RyuSat47 families, they have had enough time to accumulate within the genome, constituting 1.26% and 1.37% of the total genome, respectively (Figure 4b). Notably, we identified all 11 highly abundant, species-specific satellite families in the *M. thalassinum* genome. The MthSat170 family, devoid of recent bursts, accounted for 2.13% of the total genome. Sharing 1.6% of the total genome, the MthSat01 family exhibited two recent bursts, signifying recent genomic activity. Furthermore, a recent peak on the repeat landscape was observed for satellite families MthSat160, MthSat161, and MthSat166, comprising 1.48%, 1.54%, and 1.35%, respectively (Figure 4c). 

A similar pattern was observed within the *A. sinensis* genome, with the recently active AsiSat21 family constituting 2.06% of the genome, and old divergent residual copies of the GgrSat01, GgrSat10, GgrSat11, and MboSat26 families collectively sharing 1.8% of the total genome (Figure 4d). The species-specific satellite family exhibited newly evolved copies with recent amplification events in the *R. yunnana* genome, except for RyuSat44 with an older burst, constituting 1.54%, and EpaSat23 with a recent burst showing the conservation of this family across taxa (Figure S3a). The EpaSat02 family was recently active in the *E. pallidus* species, with four recent bursts (Figure S3b). The *P. griseoaptera* genome observed the recent burst of PgrSat01 and Pgrat02, which collectively shared 0.8% of the genome (Figure S3c). The AsiSat21 family from the ancestral *A. sinensis* species was facilitated for its proliferation by the *M. bonneti* genome and accumulated copies corresponding to 2% of the genome (Figure S3d). In our study, we also observed commonly shared satellite DNA families among some species with varying abundance and divergence from consensus sequences. Specifically, RyuSat44 satellite DNA families were found to be commonly shared between all species except *P. superangulata* and *M. thalassinum*. Likewise, AsiSat21 and MboSat26 satellite DNA families conserved across *A. sinensis* and *M. bonneti* (see Figure 4 and Figure S3). This indicates a conserved pattern of satellite DNA presence across these species.

### 3.5. Chromosomal Distribution of Satellite DNA Repeats in M. thalassinum

**Figure 5:**
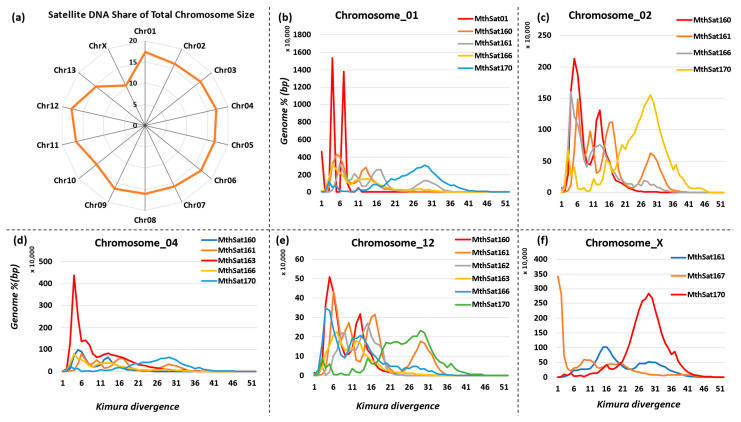
Chromosomal distribution of satellite DNA repeats. (**a**) Satellite DNA repeat abundance on chromosomes. (**b**–**f**) The satellitome landscape of highly abundant satellite DNA families on different chromosomes.

We utilized the existing chromosome-level genome assembly of *M. thalassinum* to examine the differences in satellite DNA repeat distribution across the chromosomes. The highest accumulation of satellite DNA was observed on Chr12, Chr01, and Chr04, constituting 17.79%, 17.4%, and 17.22% of the total chromosome size, respectively. Conversely, the lowest satellite abundance was identified on ChrX, accounting for 10.49% of the total size. The remaining chromosomes exhibited satellite DNA repeats ranging from a minimum of 14.76% to a maximum of 16.68% of the entire size (Figure 5a). We observed chromosome-specific propagation of satellite DNA families. For instance, MthSat01 accumulated solely on chromosome 1 with two recent bursts, constituting 1.64% of the chromosome size, while MthSat170 constituted 2.1% of the chromosome with older divergent copies (Figure 5b). In contrast, MthSat170 shared 2.33% of chromosome 2 with one older burst as well as one recent burst (Figure 5c). A similar pattern was observed for MthSat170 on chromosome 3 (Figure S4). Chromosome 4 exhibited a specific MthSat163 satellite DNA family, contributing 4% of the total chromosome size (Figure 5c). Similarly, a chromosome-5-specific MthSat162 satellite family accumulated 1.02% of the chromosome. The satellite DNA family MthSat164 was exclusively observed on chromosome 9, with an abundance of 1.18% (Figure S4). Additionally, the proliferation of the MthSat167 family was observed on chromosome X, constituting almost 1.12%. The MthSat160, MthSat161, MthSat166, and MthSat170 families have been conserved, and recently active satellite DNA families were observed across all chromosomes with a small difference in their abundance. This diversity highlights the dynamic nature of satellite DNA families and their adaptation within the genome.

## 4. Discussion

The diversity of satellite DNA repeats within the Tettigoniidae family underscores the complex genomic landscape across orthopteran species. *M. thalassinum* stands out with the highest number of satellite DNA repeats reported among insects to date, highlighting its genomic richness. Conversely, *P. griseoptera* presents the lowest number, reflecting a contrasting genomic profile. Likewise, a diverse range of satellite DNA repeat numbers is reported in many insect species [35,37,38,39]. The disparity in satellite DNA counts among congeneric species, such as *R. dubia* and *R. yunnana*, emphasizes the evolutionary divergence within the family. Similarly, species-specific satellite DNA repeats have been reported in the genus Calliptamus, which could be responsible for genome size variation [12]. Notably, satellite DNA abundance varies across species, with *R. dubia* exhibiting the highest proportion, implying functional significance or genomic expansion. Despite having fewer satellite DNA repeats, *A. sinensis* and *P. superangulata* contributes substantially to the total genome, challenging the notion that abundance solely correlates with repeat number. In *P. superangulata*, the individual PsuSat06 accounted for 4% of the total genome. A similar finding has been reported regarding the proliferation of individual satellite DNA families; for example, the ThyaSat01-301 satellite family accounted for 13.77% of the Trigona hyalinata genome [37]. This highlights the point that satellite DNA repeat numbers can influence overall abundance, as a higher number opens up the possibility of amplification within the genome, but this does not necessarily guarantee a significant impact on the overall abundance.

We found an absence of a correlation between genome size and satellite DNA families’ abundance and numbers, which suggests nuanced regulatory mechanisms shaping the genomic architecture. A similar finding has been reported indicating that satellite DNA abundance does not correlate with genome sizes in the montium group of Drosophila species [36]. However, in contrast to these findings, literature has also reported a positive correlation between genome size and satellite DNA abundance [11,40]. Satellite DNA repeats are known to be highly dynamic components of the genome [41]. In our study, we identified satellite DNA families that are commonly shared among multiple species, despite their divergence millions of years ago. Some of these species belong to the same genus, while others belong to different families. Similar conservation of satellite DNA repeats has been reported in the literature for species within the genera Gryllus, Calliptamus, and Schistocerca [12,21,42]. Additionally, a recent study on the beetle *Euchroma gigantea* reported the conservation of satellite DNA repeats across lineages [43].

Our study also reveals substantial variation in repeatome content among species, underscoring the genomic heterogeneity within the group. *M. thalassinum*, for instance, exhibits a remarkably high proportion of repetitive DNA sequences, with 92% of its genome comprising repeatome. In contrast, the outgroup species *P. superangulata* demonstrates a considerably lower repeatome content, suggesting potential regulatory differences or evolutionary distance between the outgroup and Tettigoniidae species. Our findings align with previous studies documenting the dynamic nature of TE abundance in insect genomes [44,45,46,47]. 

Our findings reveal significant variation in satellite DNA actual gain patterns across species, highlighting repetitive element proliferation and genome evolution. The observed positive trends towards amplification of species-specific satellite DNA repeats with negative divergence suggest ongoing genomic activity and adaptation within genomes [21]. Notably, species such as *R. dubia* exhibit substantial gains in satellite DNA families, indicating potential genomic expansion. Conversely, lower gains were observed in the outgroup species. Similar strategies have been applied in some studies to discern the true positive or negative change tendency of satellite DNA repeats among species [7,12,21]. 

The current study indicated notable differences in satellite DNA abundance among chromosomes, with Chr12, Chr01, and Chr04 showing the highest accumulation, while ChrX exhibited the lowest. This distribution ranged from approximately 10.49% on ChrX to around 17.79% on Chr12, highlighting the chromosome-specific nature of satellite DNA distribution. The findings align with prior literature on satellite DNA distribution in other organisms, where certain chromosomes tend to harbor higher or lower amounts of satellite repeats [6,30,48,49,50]. Furthermore, the study identified the chromosome-specific propagation of satellite DNA families, indicating distinct evolutionary dynamics at the chromosomal level. This phenomenon, where particular satellite DNA families accumulate preferentially on specific chromosomes, has been reported in other species as well [51,52]. Moreover, the specific association of certain satellite DNA families with particular chromosomes, such as MthSat163 on chromosome 4, MthSat162 on chromosome 5, and MthSat164 on chromosome 9, highlights the intricate relationship between satellite DNA evolution and chromosome structure [53,54]. These findings suggest that satellite DNA repeats exhibit non-random distribution patterns across the chromosomes of *M. thalassinum*. 

## 5. Conclusions

The comprehensive analysis of satellite DNA repeats, interspersed repeats, and satellite DNA gain and loss patterns across the Tettigoniidae family provides valuable insights into the evolutionary dynamics and genomic landscape of these orthopteran insects. Our findings underscore significant variability in repetitive element content and distribution among species, reflecting diverse adaptive strategies and evolutionary trajectories within this taxonomic group. Furthermore, the analysis of satellite DNA gain and loss patterns reveals complex patterns of amplification, homogenization, and selective propagation of satellite DNA families within genomes. These findings emphasize the role of selective pressures and adaptive mechanisms in shaping repetitive element landscapes and genomic diversity across Tettigoniidae species. The differential accumulation of satellite DNA on specific chromosomes implies potential chromosome-specific functions or structural features influencing the retention and proliferation of satellite sequences. Further research in this area is warranted to elucidate the functional significance of repetitive elements and their impact on genome structure, function, and evolution in Tettigoniidae and other insect taxa.

## Data Availability

The genomic data generated in this study have been submitted to the NCBI BioProject database (https://www.ncbi.nlm.nih.gov/search/all/?term=PRJNA763707, accessed on 14 May 2024) under accession number PRJNA763707. The library of satellite DNA families generated in this study is available on Figshare at the following (link https://doi.org/10.6084/m9.figshare.26159971.v1, accessed on 3 July 2024).

## Supplementary Materials

The following supporting information can be downloaded at https://www.mdpi.com/article/10.3390/biom14080915/s1. Figure S1: Overall gain of satellite DNA families in nine species; Figure S2: The highly abundant satellite DNA families in nine species; Figure S3: Satellitome landscape of family Tettigoniidae species; Figure S4: Chromosomal distribution of satellite DNA repeats in *M. thalassinum* species; Table S1: The detail of species and genomic data used in the current study; Table S2: Descriptive statistics of *P. superangualata* divergence and abundance; Table S3: Descriptive statistics of *R. yunnana* divergence and abundance; Table S4: Descriptive statistics of *E. pallidus* divergence and abundance; Table S5: Descriptive statistics of *P. griseoaptera* divergence and abundance; Table S6: Descriptive statistics of *R. dubia* divergence and abundance; Table S7: Descriptive statistics of *A. sinensis* divergence and abundance; Table S8: Descriptive statistics of *M. bonneti* divergence and abundance; Table S9: Descriptive statistics of *M. thalassinum* divergence and abundance; Table S10: Descriptive statistics of *G. gratiosa* divergence and abundance.
