# Supplementary material for: Evolutionary Dynamics of Satellite DNA Repeats across the Tettigoniidae Family: Insights from Genomic Analysis
Source: Biomolecules. 2024 Jul 27;14(8):915. doi: 10.3390/biom14080915 (PMC11352069; doi:10.3390/biom14080915)
Supplement: Supplementary file 1 [file biomolecules-14-00915-s001.zip › biomolecules-3039831-supplementary.pdf]

Supplementary Information

Evolutionary Dynamics of Satellite DNA Repeats Across Family Tettigoniidae: Insights from Genomic Analysis

Table S1: The detail of species and genomic data used in the current study.

Species	Family	Genome Sizes	Project Acc#	mtDNA	NCBI/Lab
Phryganogryllacris superangulata	Gryllacrididae	9.244	PRJNA607895	Yes	NCBI
Ruspolia yunnana	Tettigoniidae	9.250	PRJNA794036	Yes	NCBI
Ruspolia dubia	Tettigoniidae	9.166	PRJNA763707	Yes	Lab
Euconocephalus pallidus	Tettigoniidae	4.206	PRJNA756612	Yes	NCBI
Meconema thalassinum	Tettigoniidae	9.000	PRJEB48393	Yes	NCBI
Pholidoptera griseoaptera	Tettigoniidae	N/A	PRJNA607895	Yes	NCBI
Atlanticus sinensis	Tettigoniidae	6.801	PRJNA763707	Yes	Lab
Metrioptera bonneti	Tettigoniidae	5.494	PRJNA763707	Yes	Lab
Gampsocleis gratiosa	Tettigoniidae	7.214	PRJNA679154	Yes	NCBI

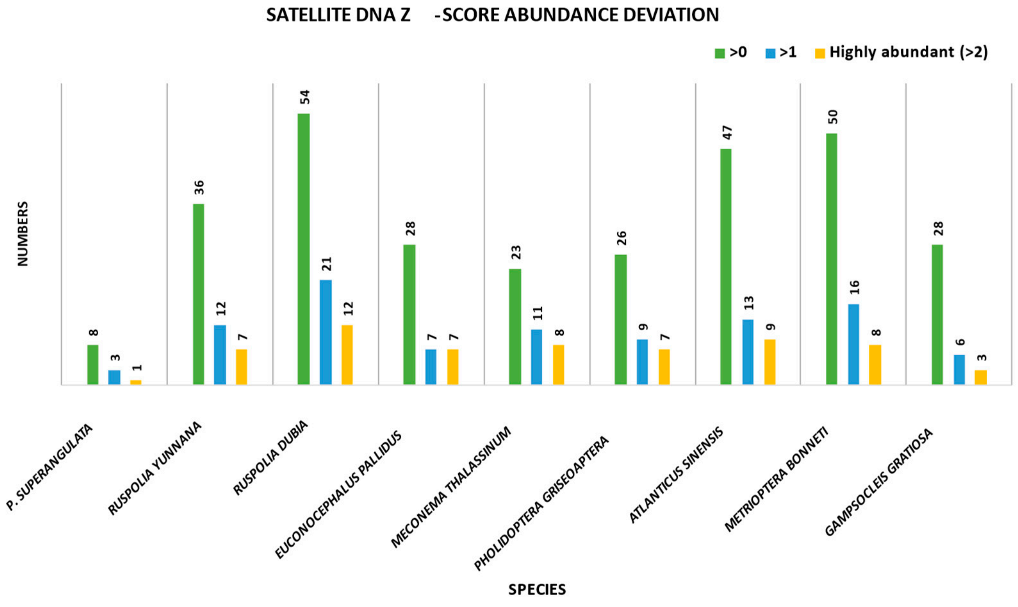

Figure S1: Overall gain of satellite DNA families in nine species. Z-score abundance and divergence were calculated for each satellite DNA family in each species. The x-axis shows the species while the y-axis depicts the total satellite DNA repeats gain (including species-specific, older residual copies from other species, and fragmental copies). The number of gain were filtered on the basis of zscore >0, >1, and >2.

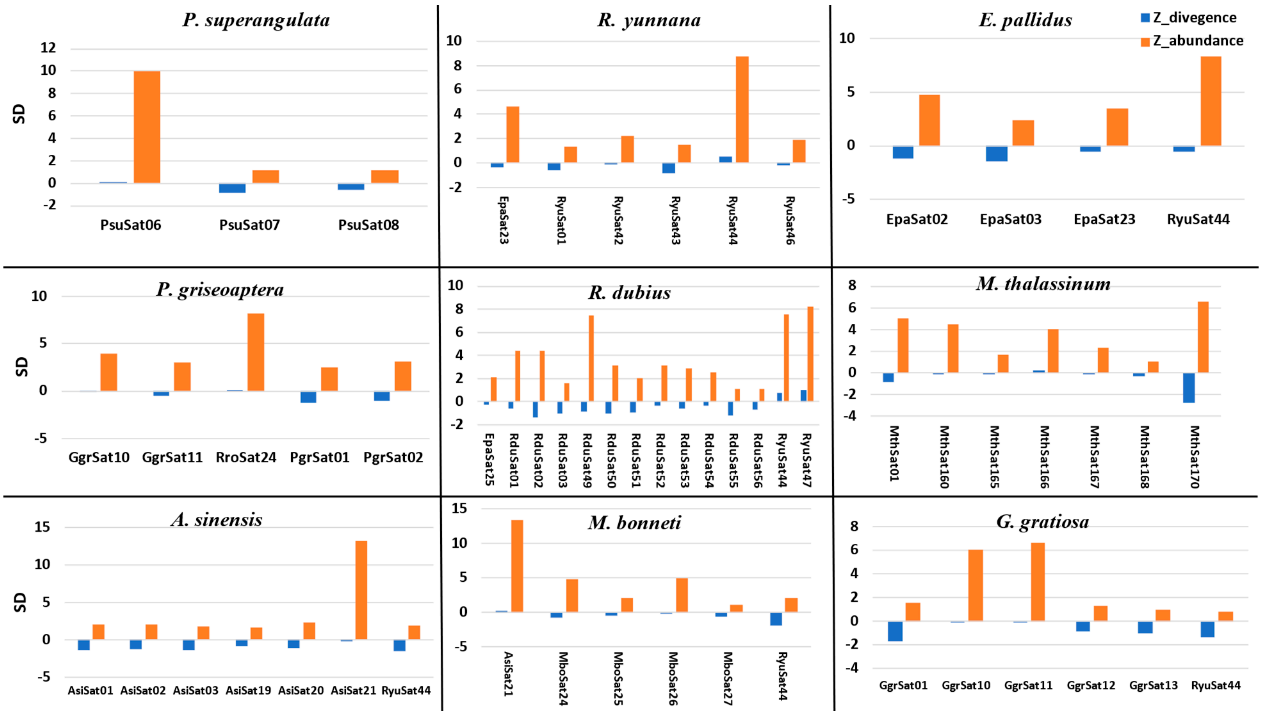

Figure S2: The highly abundant satellite DNA families in nine species. Z-score abundance positive and divergence negative satellite DNA family in each species. The x-axis shows the species while the y-axis depicts the total satellite DNA repeats gain (including species-specific, older residual copies from other species, and fragmental copies).

Table S2: Descriptive statistics of *P. superangualata* divergence and abundance

P. superangulata	**divergence**	**abundance**
**Mean**	22.68358491	0.06680317
**Standard Error**	1.0479201	0.043389757
**Median**	25.96	0.001265519

**Mode**	N/A	6.03951E-05
**Standard Deviation**	10.78899776	0.446724887
**Sample Variance**	116.4024727	0.199563125
**Range**	38.12	4.53412391
**Minimum**	0	1.80491E-05
**Maximum**	38.12	4.534141959
**Count**	106	106
**Confidence Level (95.0%)**	2.077831979	0.086033872

Table S3: Descriptive statistics of *R. yunnana* divergence and abundance

R. yunnana	**Divergence**	**Abundance**
**Mean**	14.64518987	0.068792247
**Standard Error**	0.765441611	0.013472723
**Median**	13.17	0.013521213
**Mode**	5.06	1.24808E-05
**Standard Deviation**	9.621451863	0.1693495
**Sample Variance**	92.57233595	0.028679253
**Range**	37.2	1.545538653
**Minimum**	0	1.14407E-05
**Maximum**	37.2	1.545550094
**Count**	158	158
**Confidence Level (95.0%)**	1.511891976	0.026611176

Table S4: Descriptive statistics of *E. pallidus* divergence and abundance

E. pallidus	**Divergence**	**Abundance**
**Mean**	16.85540541	0.06309545
**Standard Error**	0.776191594	0.014813122
**Median**	16.725	0.009459515
**Mode**	21.86	#N/A
**Standard Deviation**	9.442778285	0.180209407
**Sample Variance**	89.16606174	0.03247543
**Range**	37.42	1.561149435
**Minimum**	0	1.36568E-05

**Maximum**	37.42	1.561163092
**Count**	148	148
**Confidence Level (95.0%)**	1.533935682	0.029274185

Table S5: Descriptive statistics of *P. griseoaptera* divergence and abundance

P. griseoaptera	**Diveregnce**	**Abundance**
**Mean**	20.47231884	0.040954565
**Standard Error**	0.893478353	0.009552238
**Median**	21.005	0.003572771
**Mode**	21.92	N/A
**Standard Deviation**	10.49599411	0.112213392
**Sample Variance**	110.1658924	0.012591845
**Range**	41.58	0.959466582
**Minimum**	0.78	1.03362E-05
**Maximum**	42.36	0.959476918
**Count**	138	138
**Confidence Level (95.0%)**	1.766791984	0.018888894

Table S6: Descriptive statistics of *R. dubia* divergence and abundance

R. dubia	**Divergence**	**Abundance**
**Mean**	14.60733974	0.055118144
**Standard Error**	0.505261246	0.009095416
**Median**	12.485	0.008399435
**Mode**	7.09	4.54726E-06
**Standard Deviation**	8.924692991	0.160657081
**Sample Variance**	79.65014499	0.025810698
**Range**	42.8	1.374166341
**Minimum**	1.01	5.82982E-07
**Maximum**	43.81	1.374166924
**Count**	312	312
**Confidence Level (95.0%)**	0.994162705	0.017896333

Table S7: Descriptive statistics of *A. sinensis* divergence and abundance

A. sinensis	**Divergence**	**Abundance**
**Mean**	18.68923077	0.042753592

**Standard Error**	0.593344994	0.009298829
**Median**	17.35	0.001265663
**Mode**	17.12	4.39627E-06
**Standard Deviation**	9.803668232	0.15364187
**Sample Variance**	96.1119108	0.023605824
**Range**	44.89	2.066405142
**Minimum**	0.76	3.47074E-06
**Maximum**	45.65	2.066408613
**Count**	273	273
**Confidence Level(95.0%)**	1.168132438	0.018306826

Table S8: Descriptive statistics of *M. bonneti* divergence and abundance

M. bonneti	**Divergence**	**Abundance**
**Mean**	15.65854599	0.034839078
**Standard Error**	0.511184099	0.007961386
**Median**	13.79	0.000750456
**Mode**	16.81	1.86755E-05
**Standard Deviation**	9.384092636	0.146151618
**Sample Variance**	88.06119461	0.021360295
**Range**	39.21	1.995414887
**Minimum**	0	6.8744E-07
**Maximum**	39.21	1.995415574
**Count**	337	337
**Confidence Level(95.0%)**	1.005524367	0.015660439

Table S9: Descriptive statistics of *M. thalassinum* divergence and abundance

M. thalassinum	**Divergence**	**Abundance**
**Mean**	9.40791411	0.096543584
**Standard Error**	0.434504887	0.024311355
**Median**	7.88	0.001729969
**Mode**	6.33	0.000631951
**Standard Deviation**	5.547387045	0.310386602
**Sample Variance**	30.77350303	0.096339843
**Range**	32.6	2.131129833
**Minimum**	0	1.40184E-06
**Maximum**	32.6	2.131131235
**Count**	163	163
**Confidence Level(95.0%)**	0.85802366	0.048008016

Table S10: Descriptive statistics of *G. gratiosa* divergence and abundance

G. gratiosa	Divergence	Abundance
**Mean**	19.790625	0.057139339
**Standard Error**	0.828333534	0.015638782
**Median**	19.26	0.00350083
**Mode**	8.99	0.000274555
**Standard Deviation**	10.47768252	0.197816684
**Sample Variance**	109.7818311	0.03913144
**Range**	41.68	1.576921434
**Minimum**	1.05	1.04129E-05
**Maximum**	42.73	1.576931847
**Count**	160	160
**Confidence Level(95.0%)**	1.635955593	0.030886535

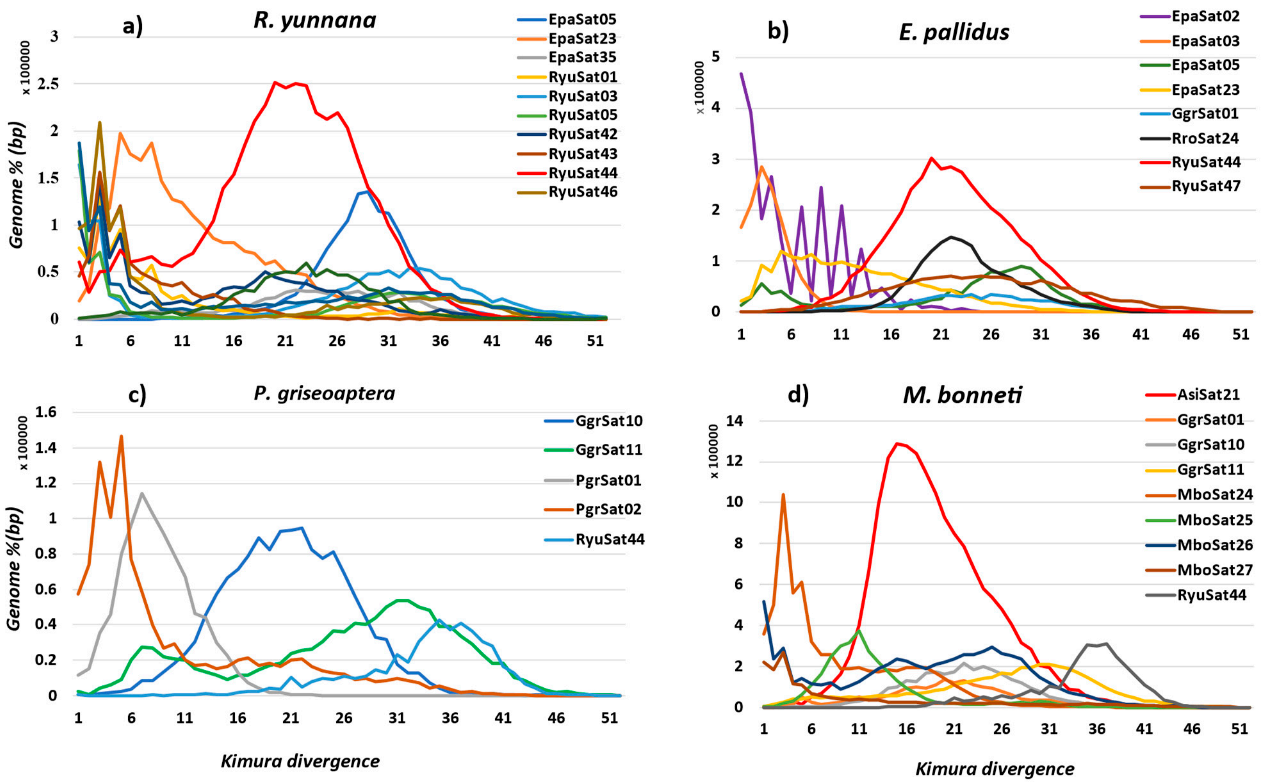

Figure S3: Satellitome landscape of family Tettigoniidae species. a) Satellite DNA repeats landscape for *R. yunnana* species. **b)** Satellite DNA repeats landscape for *E. pallidus* species. **c)** Satellite DNA repeats landscape for *P. griseoaptera* species. **d)** Satellite DNA repeats landscape for *M. bonneti* species.

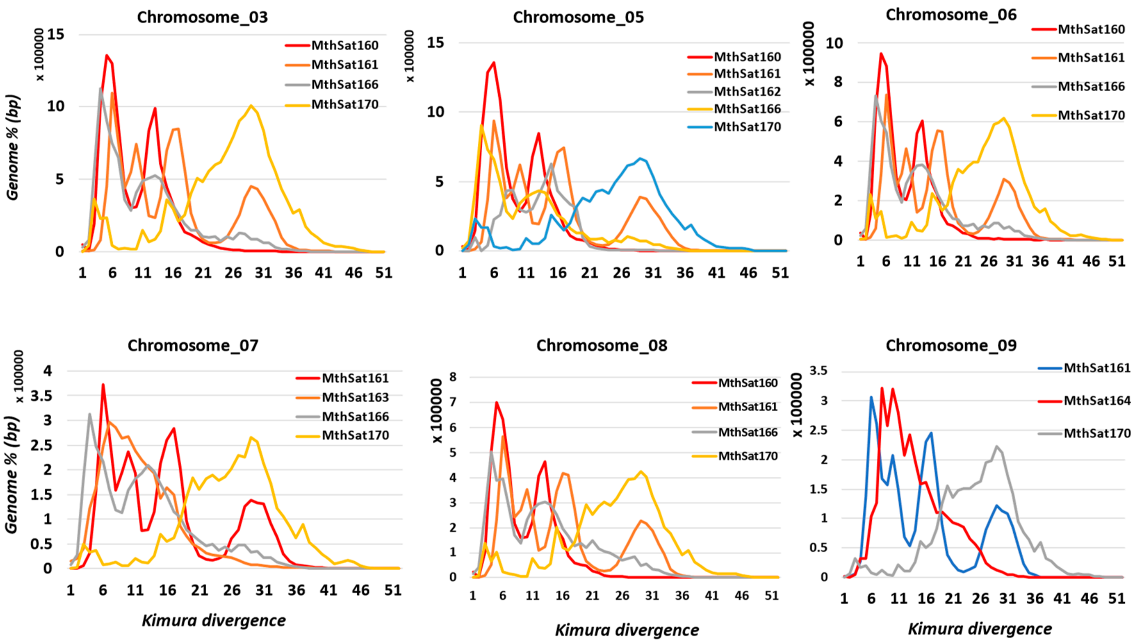

Figure S4: Chromosomal distribution of satellite DNA repeats in *M. thalassinum* species. The satellitome landscape of highly abundant satellite DNA families on different chromosomes.
